# 6-Methyl-3-phenyl-2-sulfanyl­idene-1,2,3,4-tetra­hydro­quinazolin-4-one

**DOI:** 10.1107/S1600536812007301

**Published:** 2012-02-29

**Authors:** Adel S. El-Azab, Alaa A.-M. Abdel-Aziz, Seik Weng Ng, Edward R. T. Tiekink

**Affiliations:** a Department of Pharmaceutical Chemistry, College of Pharmacy, King Saud University, Riyadh 11451, Saudi Arabia; b Department of Organic Chemistry, Faculty of Pharmacy, Al-Azhar University, Cairo 11884, Egypt; c Department of Medicinal Chemistry, Faculty of Pharmacy, University of Mansoura, Mansoura 35516, Egypt; d Department of Chemistry, University of Malaya, 50603 Kuala Lumpur, Malaysia; e Chemistry Department, Faculty of Science, King Abdulaziz University, PO Box 80203 Jeddah, Saudi Arabia

## Abstract

The title compound, C_15_H_12_N_2_OS, exists as the thione tautomer in the solid state. The phenyl group is almost perpendicular [dihedral angle = 87.96 (5)°] to the fused ring system (r.m.s. deviation = 0.036 Å for 13 ring and exocyclic non-H atoms). In the crystal, centrosymmetric dimers, sustained by pairs of N—H⋯S hydrogen bonds, are connected into layers parallel to (-101) by C—H⋯O and C—H⋯S inter­actions.

## Related literature

For recent studies on synthesis, drug discovery and crystal structures of quinazoline-4(3*H*)-one derivatives, see: El-Azab & El-Tahir (2012 ▶); El-Azab *et al.* (2011 ▶, 2010 ▶). For the anti­microbial activity of the title compound, see: Al-Omar *et al.* (2004 ▶). For the structures of related compounds, see: Bowman *et al.* (2007 ▶); Hashim *et al.* (2010 ▶).
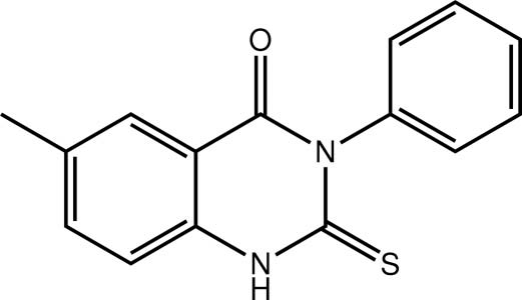


## Experimental

### 

#### Crystal data

C_15_H_12_N_2_OS
*M* *_r_* = 268.33
Monoclinic, 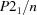

*a* = 12.7770 (3) Å
*b* = 5.1384 (1) Å
*c* = 19.0973 (4) Å
β = 91.814 (2)°
*V* = 1253.17 (5) Å^3^
*Z* = 4
Cu *K*α radiation
μ = 2.23 mm^−1^
*T* = 100 K
0.35 × 0.15 × 0.05 mm

#### Data collection

Agilent SuperNova Dual diffractometer with an Atlas detector
Absorption correction: multi-scan (*CrysAlis PRO*; Agilent, 2011 ▶) *T*_min_ = 0.967, *T*_max_ = 0.998
4636 measured reflections
2576 independent reflections
2348 reflections with *I* > 2σ(*I*)
*R*_int_ = 0.016

#### Refinement

*R*[*F* ^2^ > 2σ(*F* ^2^)] = 0.033
*wR*(*F* ^2^) = 0.098
*S* = 1.06
2576 reflections
177 parameters
H atoms treated by a mixture of independent and constrained refinement
Δρ_max_ = 0.25 e Å^−3^
Δρ_min_ = −0.25 e Å^−3^

### 

Data collection: *CrysAlis PRO* (Agilent, 2011 ▶); cell refinement: *CrysAlis PRO*; data reduction: *CrysAlis PRO*; program(s) used to solve structure: *SHELXS97* (Sheldrick, 2008 ▶); program(s) used to refine structure: *SHELXL97* (Sheldrick, 2008 ▶); molecular graphics: *ORTEP-3* (Farrugia, 1997 ▶) and *DIAMOND* (Brandenburg, 2006 ▶); software used to prepare material for publication: *publCIF* (Westrip, 2010 ▶).

## Supplementary Material

Crystal structure: contains datablock(s) global, I. DOI: 10.1107/S1600536812007301/qm2054sup1.cif

Structure factors: contains datablock(s) I. DOI: 10.1107/S1600536812007301/qm2054Isup2.hkl

Supplementary material file. DOI: 10.1107/S1600536812007301/qm2054Isup3.cml

Additional supplementary materials:  crystallographic information; 3D view; checkCIF report

## Figures and Tables

**Table 1:** Hydrogen-bond geometry (Å, °)

*D*—H⋯*A*	*D*—H	H⋯*A*	*D*⋯*A*	*D*—H⋯*A*
N2—H1*n*⋯S1^i^	0.91 (2)	2.49 (2)	3.3662 (12)	163.6 (17)
C3—H3⋯O1^ii^	0.95	2.33	3.2522 (17)	163
C11—H11⋯S1^iii^	0.95	2.86	3.7333 (16)	154
C15—H15⋯O1^iv^	0.95	2.32	3.1988 (18)	154

Symmetry codes: (i) 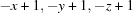; (ii) 
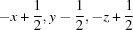
; (iii) 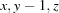; (iv) 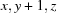.

**Fig. 1.**
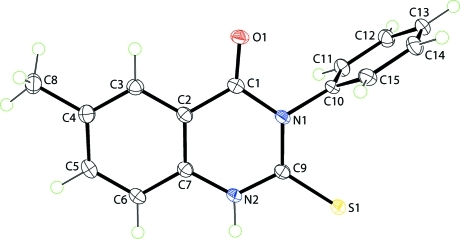
The molecular structure of (I) showing the atom-labelling scheme and displacement ellipsoids at the 50% probability level.

**Fig. 2.**
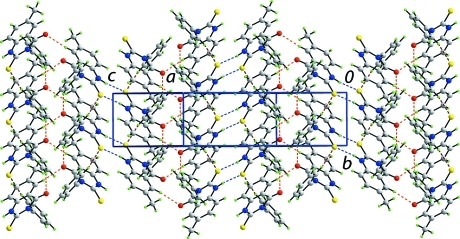
A view of the supramolecular layer parallel to (1 0 1) in (I) mediated by N—H···S, C—H···O and C—H···S interactions, shown as blue, orange and brown dashed lines, respectively.

**Fig. 3.**
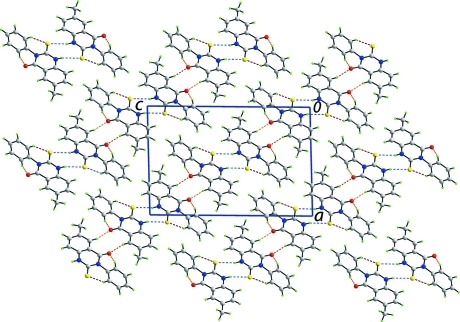
A view in projection down the b axis of the unit-cell contents of (I). The N—H···S, C—H···O and C—H···S interactions are shown as blue, orange and brown dashed lines, respectively.

## supplementary crystallographic information

### Comment

Quinazoline-4(3*H*)-one derivatives are known for their various biological activities (El-Azab & El-Tahir, 2012; El-Azab *et al.* (2011, 2010). The title compound (I) has been investigated previously for its anti-microbial activity (Al-Omar *et al.*, 2004). Herein, its crystal and molecular structure is described.

The key result of the crystal structure determination of (I), Fig. 1, is the confirmation that the compound exists as the thione tautomer in the solid-state. The non-hydrogen atoms comprising the fused ring system, including the exocyclic atoms, are co-planar with a r.m.s. deviation = 0.036 Å. The maximum deviations from the least-squares plane through these atoms are 0.038 (1) Å for the S1 atom and -0.085 (1) Å for N1, consistent with some pyramidal character for the latter atom. The phenyl group is almost perpendicular to the aforementioned plane: the dihedral angle = 87.96 (5)°.

The presence of the thione tautomer confirms the results of previous structure determinations on related compounds (Bowman *et al.*, 2007; Hashim *et al.*, 2010).

The key feature of the crystal packing is the formation of N—H···S hydrogen bonds between centrosymmetrically related molecules, Table 1. The dimeric aggregates thus formed are connected into layers parallel to (1 0 1) by C—H···O interactions, involving the bifurcated carbonyl-O atom, and C—H···S interactions, Fig. 2 and Table 1. Layers stack with no specific intermolecular interactions between them, Fig. 3.

### Experimental

A mixture of 2-amino-5-methylbenzoic acid (1.51 g m, 10 mmol) and phenyl isothiocyanate (1.35 g m, 10 mmol) in absolute ethanol (30 ml) containing triethylamine (1.1 mg, 10 mmol) was refluxed for 3 h. The reaction mixture was allowed to cool, the solvent was removed under reduced pressure, and the solid obtained was dried and recrystallized from EtOH. Yield 90%; *M*.pt: 340–342 K; ^1^H NMR (500 MHz, DMSO-d6): δ 12.98 (s, 1H, exchangeable), 7.75 (s, 1H), 7.60 (d, 1H, J=8.0 Hz), 7.48 (t, 2H, J=7.0, 7.5 Hz), 7.41 (d, 1H, J=7.0 Hz), 7.36 (d, 1H, J=8.0 Hz), 7.26 (d, 2H, J=8.0 Hz), 2.37 (s, 3H). ^13^C NMR (DMSO-d6): δ 176.0, 160.2, 139.8, 138.1, 137.1, 134.4, 129.5, 129.3, 128.5, 127.2, 116.5, 116.2, 20.9.

### Refinement

Carbon-bound H-atoms were placed in calculated positions [C—H = 0.95 to 0.98 Å, *U*_iso_(H) = 1.2 to 1.5*U*_eq_(C)] and were included in the refinement in the riding model approximation. The N—H atom was located in a difference Fourier map, and was refined with distance restraint of N—H = 0.88±0.01 Å; the *U*_iso_ value was refined.

### Crystal data

**Table:**

C_15_H_12_N_2_OS	*F*(000) = 560
*M**_r_* = 268.33	*D*_x_ = 1.422 Mg m^−^^3^
Monoclinic, *P*2_1_/*n*	Cu *K*α radiation, λ = 1.54184 Å
Hall symbol: -P 2yn	Cell parameters from 2386 reflections
*a* = 12.7770 (3) Å	θ = 3.5–76.4°
*b* = 5.1384 (1) Å	µ = 2.23 mm^−^^1^
*c* = 19.0973 (4) Å	*T* = 100 K
β = 91.814 (2)°	Prism, colourless
*V* = 1253.17 (5) Å^3^	0.35 × 0.15 × 0.05 mm
*Z* = 4	

### Data collection

**Table:**

Agilent SuperNova Dual diffractometer with an Atlas detector	2576 independent reflections
Radiation source: SuperNova (Cu) X-ray Source	2348 reflections with *I* > 2σ(*I*)
Mirror monochromator	*R*_int_ = 0.016
Detector resolution: 10.4041 pixels mm^-1^	θ_max_ = 76.6°, θ_min_ = 4.1°
ω scan	*h* = −15→16
Absorption correction: multi-scan (*CrysAlis PRO*; Agilent, 2011)	*k* = −5→6
*T*_min_ = 0.967, *T*_max_ = 0.998	*l* = −14→24
4636 measured reflections	

### Refinement

**Table:**

Refinement on *F*^2^	Primary atom site location: structure-invariant direct methods
Least-squares matrix: full	Secondary atom site location: difference Fourier map
*R*[*F*^2^ > 2σ(*F*^2^)] = 0.033	Hydrogen site location: inferred from neighbouring sites
*wR*(*F*^2^) = 0.098	H atoms treated by a mixture of independent and constrained refinement
*S* = 1.06	*w* = 1/[σ^2^(*F*_o_^2^) + (0.0613*P*)^2^ + 0.3083*P*] where *P* = (*F*_o_^2^ + 2*F*_c_^2^)/3
2576 reflections	(Δ/σ)_max_ = 0.001
177 parameters	Δρ_max_ = 0.25 e Å^−^^3^
0 restraints	Δρ_min_ = −0.25 e Å^−^^3^

### Special details

**Table:**

Geometry. All e.s.d.'s (except the e.s.d. in the dihedral angle between two l.s. planes) are estimated using the full covariance matrix. The cell e.s.d.'s are taken into account individually in the estimation of e.s.d.'s in distances, angles and torsion angles; correlations between e.s.d.'s in cell parameters are only used when they are defined by crystal symmetry. An approximate (isotropic) treatment of cell e.s.d.'s is used for estimating e.s.d.'s involving l.s. planes.
Refinement. Refinement of *F*^2^ against ALL reflections. The weighted *R*-factor *wR* and goodness of fit *S* are based on *F*^2^, conventional *R*-factors *R* are based on *F*, with *F* set to zero for negative *F*^2^. The threshold expression of *F*^2^ > σ(*F*^2^) is used only for calculating *R*-factors(gt) *etc*. and is not relevant to the choice of reflections for refinement. *R*-factors based on *F*^2^ are statistically about twice as large as those based on *F*, and *R*- factors based on ALL data will be even larger.

### Fractional atomic coordinates and isotropic or equivalent isotropic displacement parameters (Å^2^)

**Table:**

	*x*	*y*	*z*	*U*_iso_*/*U*_eq_	
S1	0.56616 (3)	0.52832 (7)	0.394981 (16)	0.01717 (12)	
O1	0.35822 (8)	−0.1192 (2)	0.25706 (5)	0.0181 (2)	
N1	0.45653 (9)	0.1596 (2)	0.32532 (6)	0.0149 (2)	
H1n	0.4357 (15)	0.305 (4)	0.4827 (11)	0.029 (5)*	
N2	0.42228 (9)	0.2138 (2)	0.44293 (6)	0.0161 (2)	
C1	0.37571 (11)	−0.0266 (3)	0.31493 (7)	0.0150 (3)	
C2	0.31942 (10)	−0.0982 (3)	0.37768 (7)	0.0156 (3)	
C3	0.24261 (10)	−0.2920 (3)	0.37432 (7)	0.0173 (3)	
H3	0.2250	−0.3722	0.3307	0.021*	
C4	0.19179 (11)	−0.3686 (3)	0.43408 (7)	0.0182 (3)	
C5	0.22073 (11)	−0.2484 (3)	0.49797 (7)	0.0195 (3)	
H5	0.1871	−0.3001	0.5394	0.023*	
C6	0.29690 (11)	−0.0568 (3)	0.50213 (7)	0.0179 (3)	
H6	0.3156	0.0210	0.5459	0.021*	
C7	0.34592 (11)	0.0206 (3)	0.44129 (7)	0.0156 (3)	
C9	0.47714 (10)	0.2888 (3)	0.38744 (7)	0.0149 (3)	
C8	0.10873 (12)	−0.5770 (3)	0.42997 (8)	0.0229 (3)	
H8A	0.1314	−0.7176	0.3993	0.034*	
H8B	0.0432	−0.5024	0.4110	0.034*	
H8C	0.0976	−0.6465	0.4769	0.034*	
C10	0.52338 (10)	0.2014 (3)	0.26589 (7)	0.0154 (3)	
C11	0.60838 (11)	0.0364 (3)	0.25902 (8)	0.0188 (3)	
H11	0.6233	−0.0942	0.2931	0.023*	
C12	0.67148 (12)	0.0652 (3)	0.20130 (8)	0.0208 (3)	
H12	0.7303	−0.0452	0.1959	0.025*	
C13	0.64818 (11)	0.2554 (3)	0.15178 (7)	0.0208 (3)	
H13	0.6910	0.2746	0.1124	0.025*	
C14	0.56249 (12)	0.4182 (3)	0.15946 (7)	0.0217 (3)	
H14	0.5471	0.5485	0.1254	0.026*	
C15	0.49894 (11)	0.3912 (3)	0.21705 (7)	0.0193 (3)	
H15	0.4400	0.5013	0.2225	0.023*	

### Atomic displacement parameters (Å^2^)

**Table:**

	*U*^11^	*U*^22^	*U*^33^	*U*^12^	*U*^13^	*U*^23^
S1	0.0194 (2)	0.01951 (19)	0.01264 (19)	−0.00399 (12)	0.00160 (13)	−0.00051 (12)
O1	0.0211 (5)	0.0210 (5)	0.0122 (5)	−0.0004 (4)	−0.0006 (4)	−0.0024 (4)
N1	0.0169 (5)	0.0178 (6)	0.0102 (5)	−0.0002 (4)	0.0019 (4)	−0.0005 (4)
N2	0.0189 (6)	0.0194 (6)	0.0101 (5)	−0.0025 (5)	0.0016 (4)	−0.0016 (5)
C1	0.0154 (6)	0.0161 (6)	0.0136 (6)	0.0029 (5)	−0.0003 (5)	0.0006 (5)
C2	0.0156 (6)	0.0179 (6)	0.0132 (6)	0.0015 (5)	0.0007 (5)	0.0012 (5)
C3	0.0168 (6)	0.0197 (7)	0.0153 (6)	0.0001 (5)	−0.0004 (5)	−0.0009 (5)
C4	0.0158 (6)	0.0190 (7)	0.0199 (7)	0.0012 (5)	0.0008 (5)	0.0013 (5)
C5	0.0193 (6)	0.0229 (7)	0.0165 (6)	0.0013 (5)	0.0052 (5)	0.0029 (6)
C6	0.0201 (7)	0.0212 (7)	0.0125 (6)	0.0008 (5)	0.0023 (5)	−0.0002 (5)
C7	0.0145 (6)	0.0176 (6)	0.0145 (6)	0.0011 (5)	0.0005 (5)	0.0002 (5)
C9	0.0155 (6)	0.0163 (6)	0.0129 (6)	0.0015 (5)	0.0002 (5)	0.0004 (5)
C8	0.0198 (7)	0.0245 (7)	0.0245 (7)	−0.0038 (6)	0.0032 (6)	0.0017 (6)
C10	0.0176 (6)	0.0183 (6)	0.0103 (6)	−0.0027 (5)	0.0026 (5)	−0.0021 (5)
C11	0.0199 (7)	0.0199 (7)	0.0166 (7)	0.0013 (5)	0.0024 (5)	0.0032 (5)
C12	0.0187 (7)	0.0225 (7)	0.0213 (7)	0.0011 (6)	0.0056 (6)	−0.0005 (6)
C13	0.0230 (7)	0.0249 (7)	0.0148 (6)	−0.0055 (6)	0.0060 (5)	−0.0011 (6)
C14	0.0283 (8)	0.0220 (7)	0.0148 (7)	−0.0007 (6)	0.0024 (6)	0.0051 (6)
C15	0.0226 (7)	0.0192 (7)	0.0161 (6)	0.0025 (6)	0.0024 (5)	−0.0008 (6)

### Geometric parameters (Å, º)

**Table:**

S1—C9	1.6788 (14)	C6—C7	1.3953 (19)
O1—C1	1.2175 (17)	C6—H6	0.9500
N1—C9	1.3776 (17)	C8—H8A	0.9800
N1—C1	1.4171 (18)	C8—H8B	0.9800
N1—C10	1.4578 (16)	C8—H8C	0.9800
N2—C9	1.3454 (17)	C10—C15	1.379 (2)
N2—C7	1.3913 (18)	C10—C11	1.388 (2)
N2—H1n	0.91 (2)	C11—C12	1.3940 (19)
C1—C2	1.4639 (18)	C11—H11	0.9500
C2—C7	1.3918 (19)	C12—C13	1.386 (2)
C2—C3	1.398 (2)	C12—H12	0.9500
C3—C4	1.3877 (19)	C13—C14	1.389 (2)
C3—H3	0.9500	C13—H13	0.9500
C4—C5	1.406 (2)	C14—C15	1.3945 (19)
C4—C8	1.508 (2)	C14—H14	0.9500
C5—C6	1.385 (2)	C15—H15	0.9500
C5—H5	0.9500		
			
C9—N1—C1	124.38 (11)	N2—C9—N1	116.73 (12)
C9—N1—C10	119.89 (11)	N2—C9—S1	120.73 (10)
C1—N1—C10	115.66 (11)	N1—C9—S1	122.54 (10)
C9—N2—C7	124.69 (12)	C4—C8—H8A	109.5
C9—N2—H1n	115.0 (13)	C4—C8—H8B	109.5
C7—N2—H1n	120.2 (13)	H8A—C8—H8B	109.5
O1—C1—N1	120.20 (12)	C4—C8—H8C	109.5
O1—C1—C2	124.34 (13)	H8A—C8—H8C	109.5
N1—C1—C2	115.45 (12)	H8B—C8—H8C	109.5
C7—C2—C3	120.25 (12)	C15—C10—C11	121.98 (12)
C7—C2—C1	119.46 (13)	C15—C10—N1	120.36 (12)
C3—C2—C1	120.24 (12)	C11—C10—N1	117.59 (12)
C4—C3—C2	120.66 (13)	C10—C11—C12	118.93 (13)
C4—C3—H3	119.7	C10—C11—H11	120.5
C2—C3—H3	119.7	C12—C11—H11	120.5
C3—C4—C5	118.17 (13)	C13—C12—C11	119.84 (14)
C3—C4—C8	120.35 (13)	C13—C12—H12	120.1
C5—C4—C8	121.48 (13)	C11—C12—H12	120.1
C6—C5—C4	121.79 (13)	C12—C13—C14	120.37 (13)
C6—C5—H5	119.1	C12—C13—H13	119.8
C4—C5—H5	119.1	C14—C13—H13	119.8
C5—C6—C7	119.22 (13)	C13—C14—C15	120.25 (13)
C5—C6—H6	120.4	C13—C14—H14	119.9
C7—C6—H6	120.4	C15—C14—H14	119.9
N2—C7—C2	118.93 (12)	C10—C15—C14	118.62 (13)
N2—C7—C6	121.17 (13)	C10—C15—H15	120.7
C2—C7—C6	119.90 (13)	C14—C15—H15	120.7
			
C9—N1—C1—O1	175.07 (13)	C5—C6—C7—N2	179.51 (13)
C10—N1—C1—O1	−7.96 (18)	C5—C6—C7—C2	−1.2 (2)
C9—N1—C1—C2	−6.19 (19)	C7—N2—C9—N1	−1.3 (2)
C10—N1—C1—C2	170.77 (11)	C7—N2—C9—S1	178.88 (10)
O1—C1—C2—C7	−179.92 (13)	C1—N1—C9—N2	6.2 (2)
N1—C1—C2—C7	1.40 (19)	C10—N1—C9—N2	−170.65 (12)
O1—C1—C2—C3	2.7 (2)	C1—N1—C9—S1	−173.97 (10)
N1—C1—C2—C3	−176.00 (12)	C10—N1—C9—S1	9.18 (18)
C7—C2—C3—C4	0.0 (2)	C9—N1—C10—C15	−91.74 (16)
C1—C2—C3—C4	177.40 (13)	C1—N1—C10—C15	91.15 (16)
C2—C3—C4—C5	−0.9 (2)	C9—N1—C10—C11	91.23 (16)
C2—C3—C4—C8	179.76 (13)	C1—N1—C10—C11	−85.88 (15)
C3—C4—C5—C6	0.6 (2)	C15—C10—C11—C12	0.6 (2)
C8—C4—C5—C6	−179.97 (13)	N1—C10—C11—C12	177.61 (13)
C4—C5—C6—C7	0.4 (2)	C10—C11—C12—C13	−0.5 (2)
C9—N2—C7—C2	−3.2 (2)	C11—C12—C13—C14	0.2 (2)
C9—N2—C7—C6	176.10 (13)	C12—C13—C14—C15	−0.2 (2)
C3—C2—C7—N2	−179.69 (12)	C11—C10—C15—C14	−0.6 (2)
C1—C2—C7—N2	2.9 (2)	N1—C10—C15—C14	−177.45 (13)
C3—C2—C7—C6	1.0 (2)	C13—C14—C15—C10	0.3 (2)
C1—C2—C7—C6	−176.36 (12)		

### Hydrogen-bond geometry (Å, º)

**Table:**

*D*—H···*A*	*D*—H	H···*A*	*D*···*A*	*D*—H···*A*
N2—H1*n*···S1^i^	0.91 (2)	2.49 (2)	3.3662 (12)	163.6 (17)
C3—H3···O1^ii^	0.95	2.33	3.2522 (17)	163
C11—H11···S1^iii^	0.95	2.86	3.7333 (16)	154
C15—H15···O1^iv^	0.95	2.32	3.1988 (18)	154

Symmetry codes: (i) −*x*+1, −*y*+1, −*z*+1; (ii) −*x*+1/2, *y*−1/2, −*z*+1/2; (iii) *x*, *y*−1, *z*; (iv) *x*, *y*+1, *z*.
